# Overall survival and progression-free survival in pediatric meningiomas: a systematic review and individual patient-level meta-analysis

**DOI:** 10.1007/s11060-024-04917-7

**Published:** 2025-01-09

**Authors:** Johannes Wach, Martin Vychopen, Alim Emre Basaran, Marcos Tatagiba, Roland Goldbrunner, Erdem Güresir

**Affiliations:** 1 Department of Neurosurgery, University Hospital Leipzig, Leipzig University, Liebigstraße, 20, 04103, Leipzig, Germany; 2 Comprehensive Cancer Center Central Germany, Partner Site Leipzig, Leipzig, Germany; 3 Center for Neuro-Oncology, Comprehensive Cancer Center Tübingen-Stuttgart, University Hospital Tübingen, Eberhard Karls University Tübingen, Tübingen, Germany; 4 Department of Neurosurgery, University Hospital Tübingen, Eberhard Karls University Tübingen, Tübingen, Germany; 5 Department of General Neurosurgery, Center of Neurosurgery, University of Cologne, Cologne, Germany

**Keywords:** Pediatric Meningiomas, Overall Survival, Progression-Free Survival, WHO Tumor Grade, Extent of resection

## Abstract

**Background:**

Pediatric meningiomas (PMs) are rare central nervous system tumors, accounting for 1–5% of all meningiomas, and differ from adult meningiomas in clinical, histopathological, and molecular features. Current guidelines primarily focus on adults, leaving a gap in evidence-based management for PMs. This study presents the largest meta-analysis of longitudinal individual patient data (IPD) to date, addressing progression-free survival (PFS) and overall survival (OS) in pediatric patients.

**Methods:**

Data from 20 studies (2011–2023), including 1010 pediatric meningioma cases, were analyzed to assess PFS and OS stratified by WHO grade, NF1/NF2 status, extent of resection (EOR), and adjuvant radiotherapy. Longitudinal survival data were reconstructed from Kaplan–Meier curves using IPD extraction methods.

**Results:**

PMs affect males and females nearly equally (52.1% vs. 47.9%). WHO grade 3 tumors had significantly shorter PFS (72.1 months) compared to grades 1 (209.8 months) and 2 (137.5 months) (*p* < 0.001). No significant OS difference between WHO grades 1 and 2 PMs were observed. NF1- and NF2-associated tumors showed shorter PFS (59.7 and 138.4 months) than sporadic cases (180.6 months) (*p* = 0.02). GTR significantly improved PFS (113.8 vs. 40.1 months, *p* < 0.001) and OS (602.9 vs. 173.8 months, *p* < 0.001). Radiotherapy enhanced PFS (72.5 vs. 23.8 months, *p* = 0.009) and OS (140.7 vs. 63.0 months, *p* = 0.002) in grade 3 tumors but not in WHO grade 2 PMs (*p* = 0.43).

**Conclusions:**

This largest meta-analysis highlights the critical roles of GTR and adjuvant radiotherapy in improving outcomes for high-grade PMs and underscores the urgent need for pediatric-specific management guidelines based on robust longitudinal data.

**Graphical Abstract:**

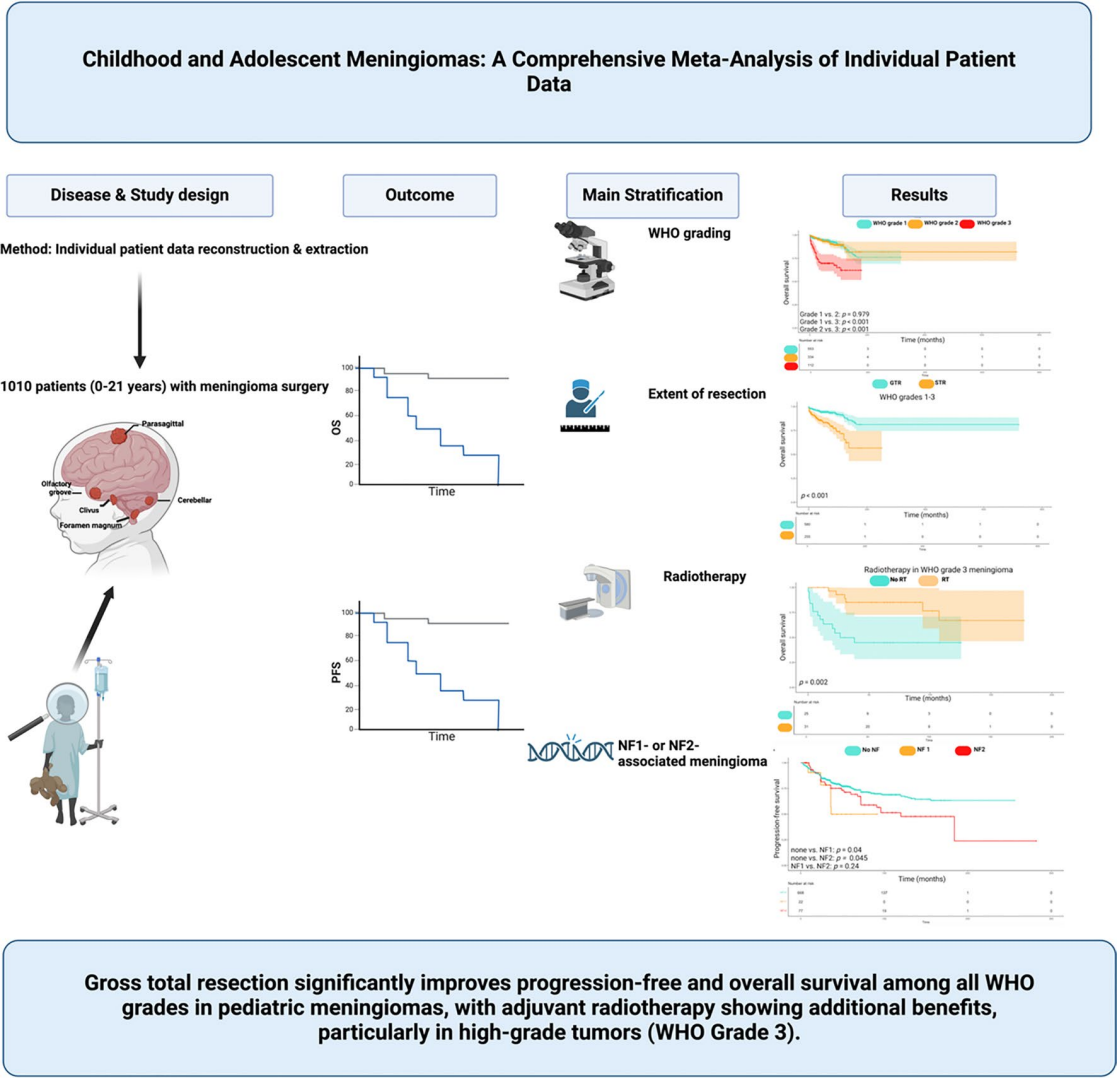

**Supplementary Information:**

The online version contains supplementary material available at 10.1007/s11060-024-04917-7.

## Introduction

Pediatric meningiomas (PMs), though rare in the central nervous system (CNS), present unique clinical challenges distinct from their adult counterparts. Meningiomas are the most frequently observed primary CNS tumors in adults, and advancements in genetic and epigenetic characterizations have significantly improved understanding of their management [1–3]. However, PMs, which account for only 1–5% of all meningiomas, differ clinically, histopathologically, and molecularly from adult meningiomas [4, 5].

The last comprehensive meta-analysis on PMs was published in 2011, highlighting the need for updated research [6]. Current meningioma guidelines, such as those from EANO, primarily focus on adults, creating a gap in management strategies for PMs [7]. PMs exhibit distinct clinical features, including a higher prevalence of clear cell subtypes and different genetic mutations compared to adults [4]. In PMs, neurofibromatosis (NF) 2 mutations predominate, while genetic alterations such as TRAF7, AKT1, and SMO are uncommon [8–11].

Given these differences, it is crucial to investigate the factors influencing progression-free survival (PFS) and overall survival (OS) in PMs. This pooled meta-analysis aims to inform clinical practice guidelines tailored for PMs.

## Methods

### Search strategy and data collection

This meta-analysis adhered to the PRISMA checklist (see Supplementary Methods 1) and was prospectively registered in the International Prospective Register of Systematic Reviews (ID: CRD42024601057) [12]. Individual patient datasets (IPDs) were extracted from PubMed, Google Scholar, and Cochrane library between January 1, 2011, and August 30, 2024 (see Supplementary Table 1). The search utilized both MeSH and non-MeSH keywords, including "meningioma," "child," "adolescent," "infant," and "pediatric". The study protocol is given in Supplementary Methods 2.

### Inclusion criteria

Inclusion was limited to studies in English involving a minimum of three patients with longitudinal follow-up data (PFS or OS), focusing on patients aged 21 or younger with histopathologically confirmed cranial sporadic or NF-associated meningiomas.

### Quality assessment

Methodological quality and bias were assessed using the NIH Quality Assessment Tool (NIH-QAT) [13], providing a systematic evaluation of study strengths and limitations.

### Data extraction

Two authors (AB, JW) independently extracted data (age, neuroanatomical localization, sex, sporadic or NF, extent of resection (EoR), chemotherapy or radiotherapy). PFS data and numbers at risk were extracted from Kaplan–Meier survival curves in Jagtiani et al. [14] and Kotecha et al. [6] using Digitizelt software (Version 2.5.10 for macOS) and reconstructed with the R package IPDfromKM [15, 16]. EoR was categorized as gross total resection (GTR, Simpson grades I-III) or subtotal resection (STR, Simpson grade > III). Data on dural attachment treatment were not available, limiting this analysis. Discrepancies between authors were resolved through re-examination or consultation with a third author (EG). Table 1 provides an overview of the included studies and their patients with matching criteria for extracting data. Additional data were retrieved from manuscripts or Supplementary Data [17–34].

**Table 1:** Patient characteristics of the 20 included studies with proportions of their eligible patients with follow-up data

Study	Country	Number of included patients	Sex (M:F)	Median Age (Interquartile range)	Neuroanatomical Location	NF1	NF2	GTR	STR	Radio-therapy	Chemo-therapy
Jagtiani et al., 2024 [14]	USA	239	117:122	N/A	Intracranial (Not further specified)	N/A	N/A	76/118	42/118	65/231	N/A
Gader et al., 2024 [17]	Tunisia	3	1:2	3 (2–9.5)	Right ponto-cerebellar angle (1) Left occipital (1) Left parietal (1)	0/3	0/3	3/3	0/3	0/3	0/3
García-Marqueta et al., 2023 [18]	Switzerland	9	7:2	13 (6–14)	Skull base (7) Optic nerve sheath (1) Convexity (1)	0/9	0/9	0/9	9/9	9/9	0/9
Chen et al., 2023 [19]	China	5	4:1	7 (6–11)	Lateral ventricle (5)	0/5	0/5	5/5	0/5	0/5	0/5
Opoku et al., 2022 [20]	China	10	6:4	9.5 (4.75–13.5)	Left parietal lobe (1) Right cerebellum (1) Third ventricle (1) Right lateral ventricle (2) Right parasellar (1) Left lateral ventricle (2) Left temporal lobe (1) Foramen magnum (1)	0/10	0/10	10/10	0/10	0/10	0/10
Santana-González et al., 2021 [21]	Mexico	19	11:8	12.5 (8.25–14)	Supra and infra sellar (1) Parietal lobe (1) Left side Optic nerve (2) Interhemispheric tumor (1) Left Occipital (1) Right parieto-temporal (1) Intraventricular (2) Right frontal lobe (1) Pineal region (2) Left fronto-parietal (1) Left fronto-parieto-temporal (1) Rigth parietal parasagittal (1) Left Parieto-temporal (1) Bulb (1) Right occipito-parietal (1) Suprasellar retrochiasmatic (1)	1/19	2/19	EoR known for 15/19 patients: 5/15	10/15	N/A	N/A
Liu et al., 2021 [22]	China	39	18:21	11 (8–14)	Intraventricular (11) Convexity (15) Skull base (6) Posterior fossa (4) Parasagittal/ falx (3)	0/39	0/39	32/39	7/39	2/39	0/39
Fouda et al., 2021 [23]	USA	7	2:5	5 (2.5–10)	Posterior fossa (2) Sylvian fissure (1) Middle fossa (2) Skull base (2)	0/7	0/7	3/7	4/7	N/A	N/A For all radiation-induced meningiomas (11/
He et al., 2020 [24]	China	31	18:13	13 (8.5–14.5)	Cranio-orbital (1) Convexity (11) Lateral ventricle (5) Sphenoid ridge (3) Cerebello pontine angle (2) Middle cranial fossa (1) Foramen magnum (1) Optic nerve (1) Parasagittal (3) Sellar region (1) Petroclival (2)	2/31	1/31	21/31	10/31	7/31	NA
Toland et al., 2020 [11]	USA	40	24:16	13 (10–16)	Convexity (21) Skull base (16) NA (3)	0/40	12/40	N/A	N/A	N/A	N/A
Amirjamshidi et al., 2019 [25]	Iran	3	1:2	7 (6–7)	Sylvian fissure (3)	0/3	0/3	3/3	0/3	0/3	0/3
Tauziede-Espariat et al., 2018 [26]	France	4	1:3	10 (6.75–14.25)	Petrous and clivus (1) Right temporal convexity (1) Petrous (1) Petrous and cavernous sinus (1)	0/4	0/4	3/4	1/4	0/4	0/4
Liu et al., 2017 [27]	China	19	9:10	16 (13.5–17.5)	Left pertroclival region (2) Right petroclival region and CPA (1) Right CPA (5) Left CPA (1) Left cerebellum (1) Anteriolateral foramen magnum (4) Lateral foramen magnum (1) Posterior foramen magnum (1) Anterior foramen magnum (1) Foramen magnum to C2 (1) Left jugular foramen (1)	0/19	2/19	13/19	6/19	7/19	0/19
Dash et al., 2016 [28]	India	6	4:2	16 (12.75–17.75)	Left trigone (1) Left atrium and occipital horn (1) Right lateral ventricle (1) Third ventricle (1) Right trigone (1) Left lateral ventricle (1)	0/6	1/6	6/6	0/6	0/6	NA
Donovan et al., 2016 [29]	USA	3	2:1	11 (9–13.5)	Sylvian fissure (3)	0/3	0/3	1/3	2/3	1/3	0/3
Burkhardt et al., 2013 [30]	Switzerland	12	7:5	13 (10.25–14.5)	Olfactory groove (1) Left sphenoid wing (1) Tuberculum sellae (4) Right parietal parasagittal (1) Left parietal parasagittal (2) Right frontal convexity (1) Left frontal convexity (2)	0/12	2/12	3/12	9/12	3/12	NA
Wang et al., 2012 [31]	China	20	10:10	13 (9–16.25)	Right occipital parasagittal (1) Right parietal (1) Left CPA (4) Anterior cranial fossa (1) Left occipital (1) Left frontal (2) C4-5 (1) Left sphenoidal crest (1) C1-2 (1) C6-T2 (1) Corpus callosum (1) Right occipital (1) Left frontal–temporal (1) Bilateral frontal (1) Right CPA (1) Left temporal (1)	0/20	3/20	6/20	14/20	Unknown for each individual patient	NA
Santos et al., 2012 [32]	Brazil	14	8:6	14 (11.5–17)	Left sphenoidal (1) Falcotentorial (1) Right temporal base/ petrous apex (1) Right parietal parasagittal (4) Right parietal (1) Left jugular foramen (1) Righ frontoparietal parasagittal (1) Left frontoparietal (1) Intraventricular (1) Left parasagittal/ sphenoidal (1) Right sphenoidal (1)	0/15	5/15	12/15	3/15	4/15	NA
Jaiswal et al., 2011 [33]	India	8	6:2	17 (13.5–18)	Left frontal (1) Right lateral ventricle (1) Left frontal (2) Left tentorial (posterior fossa) (1) Left falco-tentorial (1) Right pterional (1) Right temporal (1)	0/8	0/8	6/8	2/8	5/8	NA
Kotecha et al., 2011 [6]	Australia	519	299:248 (for total cohort with 28 additional spinal cases which are not included in this analysis)	N/A	Supratentorial (455) Infratentorial (64)	20/547	49/547	395/547	152/547	52/547	N/A

*F* Female, *GTR* Gross Total Resection, *M* Male, *N/ A* not assessed, *NF* Neurofibromatosis, *STR* Subtotal Resection

### Statistical analysis

Pooled IPD were used to construct Kaplan–Meier curves for OS and PFS, stratified by age, WHO grade, neurofibromatosis status, EoR, and adjuvant radiotherapy. Treatment characteristics, including EoR and radiotherapy, were further stratified by WHO grade. A subgroup analysis IPD was conducted for the cohorts created by direct data extraction with available multiple variables. Within this subgroup, 184 patients had common covariates, including age, sex, WHO grade, EoR, and conduction of radiation therapy. Uni- and multivariable Cox regression analysis was performed on this subset to identify independent factors predicting meningioma progression in each WHO grade. Analyses were performed using the R packages ‘Survminer’ and ‘Survival’ (v4.3.1, R Foundation). Subgroup comparisons for PFS and OS employed the log-rank test (*p* < 0.05).

## Results

### Search results and included studies

**Fig. 1:**
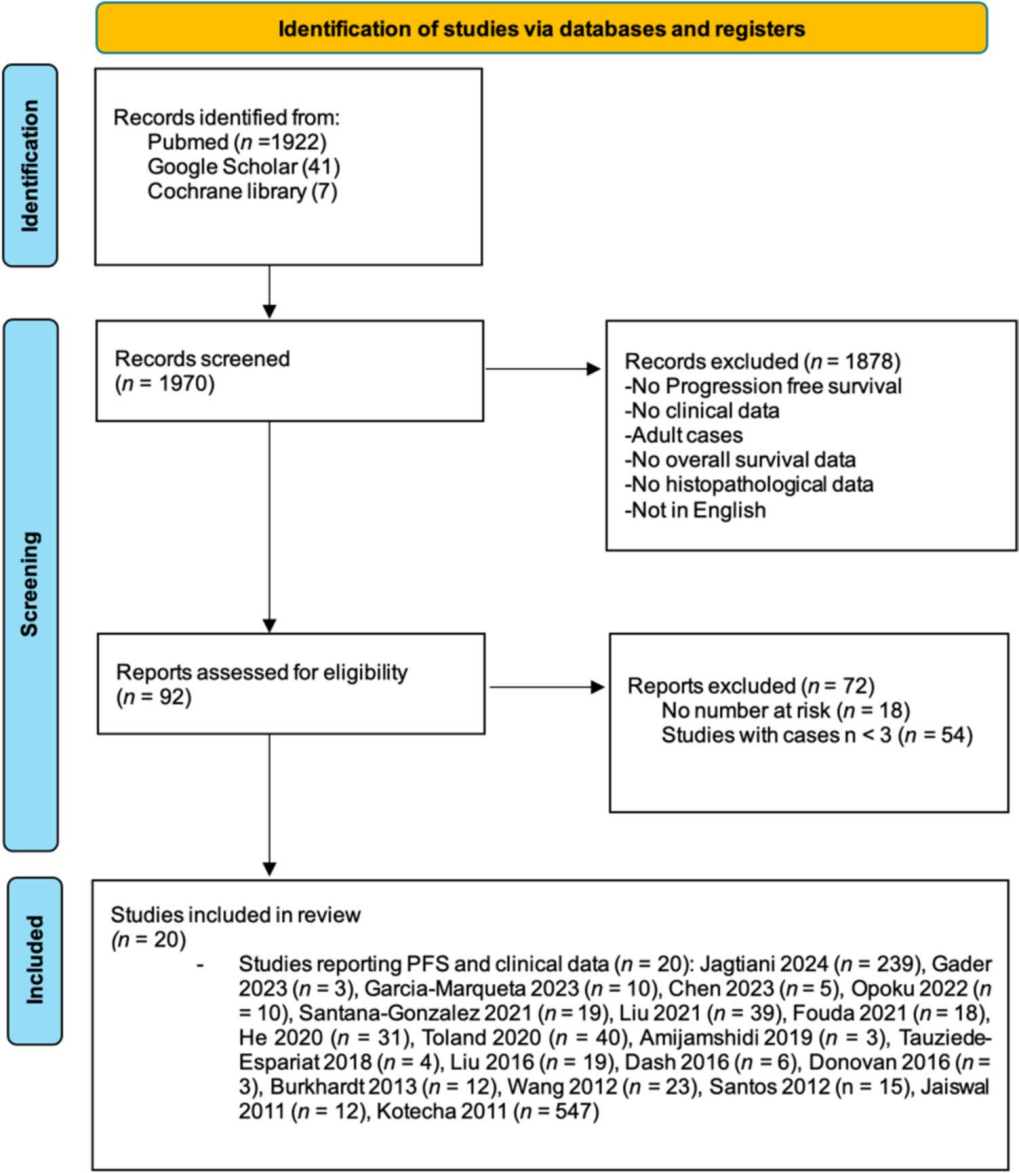
PRISMA flowchart for study selection

The initial search identified 1970 studies; 1878 were excluded based on titles and abstracts. Of the remaining 92, 72 lacked sufficient PFS data or had fewer than three patients, leaving 20 studies for meta-analysis (Fig. 1). Published between 2011 and 2024, these included six from China, four from the USA, two each from India and Switzerland, and one each from Tunisia, Mexico, Iran, France, Brazil, and Australia. Unlike adults, PMs show a nearly equal male-to-female distribution (52.1% males, 47.9% females). The NF status and WHO grade distribution were analyzed in 252 patients. Among WHO grade 1 meningiomas, 86.2% were sporadic, 1.4% had NF type 1, and 12.4% had NF type 2, representing 57.5% of the total cohort. WHO grade 2 meningiomas included 88.5% without NF and 11.5% with NF type 2, comprising 31.0% of the cohort, while grade 3 meningiomas were sporadic in 89.7% and 10.3% with NF type 2, making up 11.5%. Overall, most patients across all WHO grades had sporadic tumors (87.3%), with NF2 cases relatively consistent across WHO grades. No detailed IPD on adjuvant chemotherapy was available. Nineteen studies were retrospective single-center analyses [14–33], and one was a meta-analysis [6] (Table 1).

### Progression-free survival and overall survival in pediatric and adolescent meningioma

**Fig. 2:**
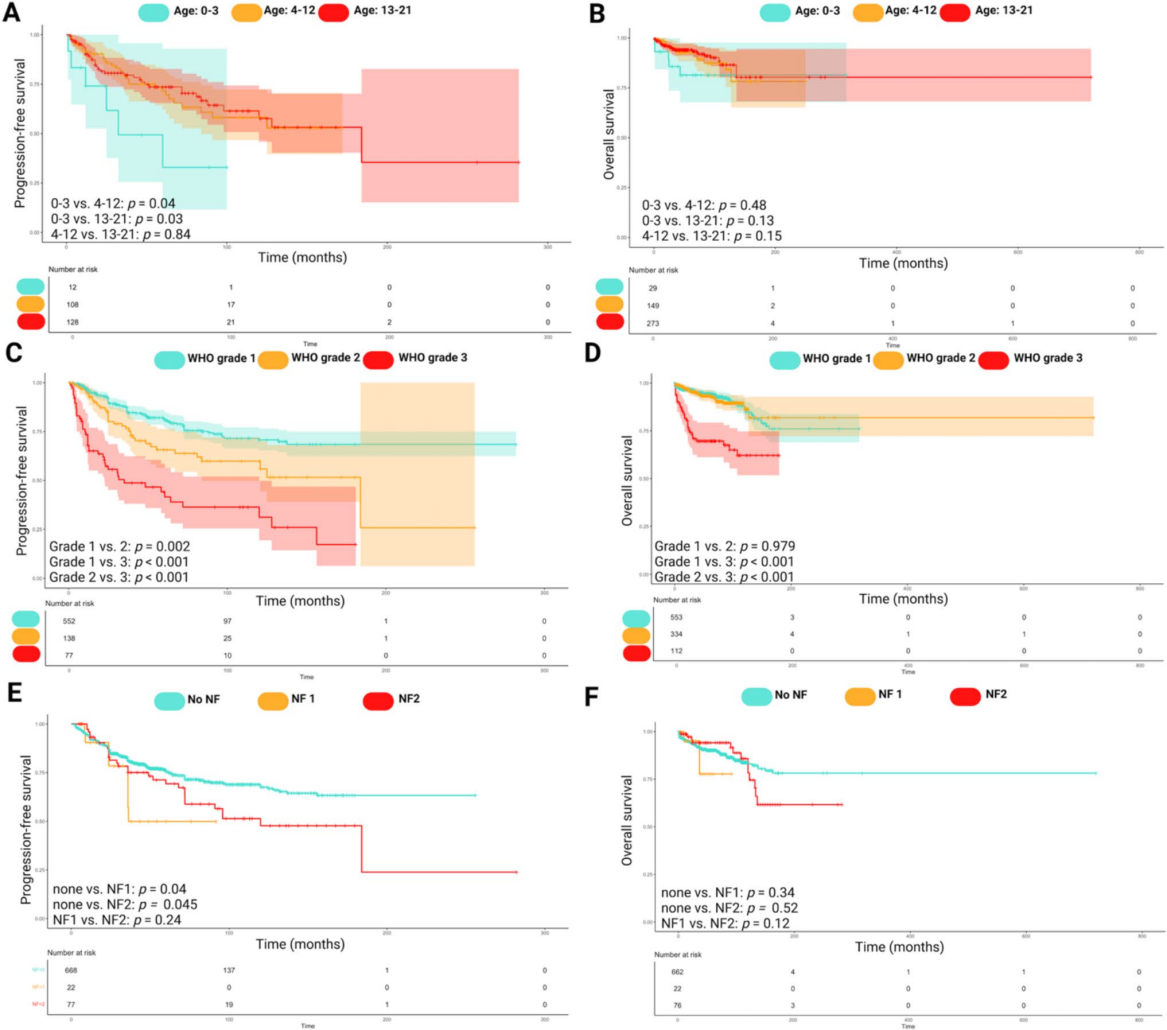
Kaplan–Meier survival analysis for progression-free survival (PFS) and overall survival (OS) based on age, WHO tumor grade, and neurofibromatosis type (NF1/NF2) status. **A**, **B**: PFS and OS stratified by age groups: 0–3 years (blue), 4–12 years (orange), and 13–21 years (red). Statistical significance between age groups is shown in each panel. **C**, **D**: PFS (**C**) and OS (**D**) based on WHO tumor grade: Grade 1 (blue), Grade 2 (orange), and Grade 3 (red). Significant differences between the WHO grades are highlighted. **E**, **F**: PFS (**E**) and OS (**F**) stratified by neurofibromatosis status: No NF (blue), NF1 (orange), and NF2 (red). *p*-values between groups are displayed, indicating statistical comparisons. The number of patients at risk for each time point is provided below each panel. Shaded areas represent 95% confidence intervals

Mean PFS for children diagnosed with meningioma at ages 0–3, 4–12, and 13–21 were 51.3 months (95%CI: 26.4–76.2), 115.1 months (95%CI: 99.2–130.9), and 158.9 months (95%CI: 121.7–196.1), respectively. PFS was significantly shorter in those diagnosed at 0–3 years compared with 4–12 years (*p* = 0.04) and 13–21 years (*p* = 0.03, Fig. 2a). OS stratified by age groups showed no significant differences: 261.7 months (95%CI: 217.8–305.6) for 0–3 years, 212.3 months (95%CI: 191.6–233.0) for 4–12 years, and 594.5 months (95%CI: 516.1–672.8) for 13–21 years (*p* = 0.29, Fig. 2b).

### Progression-free survival and overall survival among WHO grades in pediatric and adolescent meningioma

WHO grading strongly differentiated PMs regarding PFS. Mean PFS for WHO grades 1, 2, and 3 were 209.8 months (95%CI: 197.0–222.7), 137.5 months (95%CI: 104.3–170.8), and 72.1 months (95%CI: 53.7–90.5), respectively (*p* < 0.001, Fig. 2c). OS showed no significant difference between grades 1 and 2 (*p* = 0.98). Five-, 10-, and 15-year OS probabilities for grade 1 were 94.3%, 87.3%, and 76.0%; for grade 2, 93.4%, 86.4%, and 81.9%; and for grade 3, 69.7% and 62.2% at five and 10 years. OS for grade 3 was significantly shorter than grades 1 (*p* < 0.001) and 2 (*p* < 0.001, Fig. 2d).

### Progression-free survival and overall survival among sporadic or NF-associated meningiomas in children and adolescents

Among 767 patients, mean PFS for sporadic, NF1-, and NF2-associated PMs were 180.6 months (95%CI: 170.3–191.0), 59.7 months (95%CI: 43.6–75.8), and 138.4 months (95%CI: 97.5–179.5), respectively (*p* = 0.02, Fig. 2e). NF1- (*p* = 0.04) and NF2-associated PMs (*p* = 0.045) had significantly shorter PFS than sporadic PMs. OS did not differ significantly, with a median follow-up of 51.7 months (IQR: 23.1–100.2, Fig. 2f).

### Progression-free survival and overall survival by extent of resection in pediatric meningioma

**Fig. 3:**
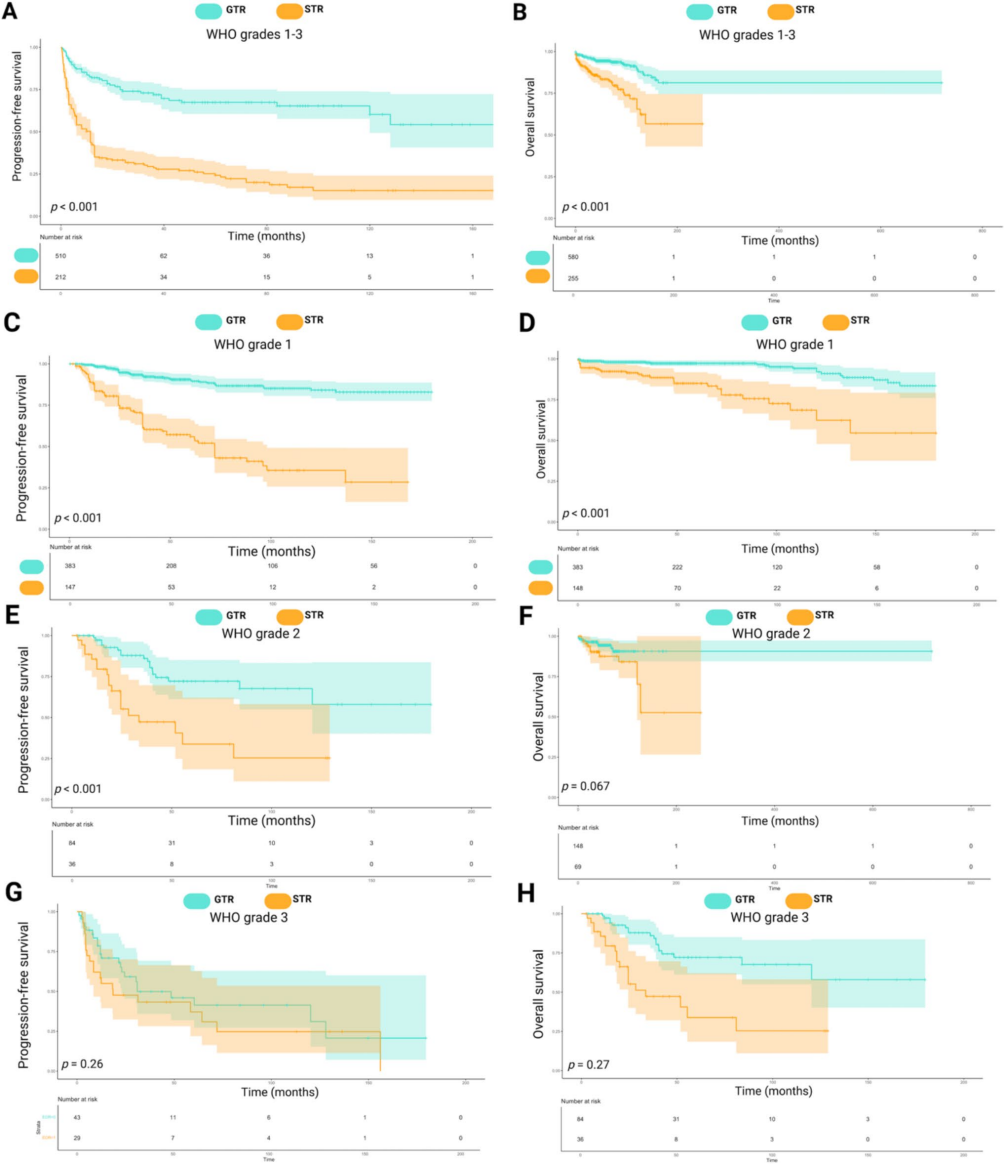
Kaplan–Meier survival analysis for progression-free survival (PFS) and overall survival (OS) based on the extent of resection (GTR: Gross Total Resection, STR: Subtotal Resection) and WHO tumor grades. **A**, **B**: PFS (**A**) and OS (**B**) for WHO Grades 1–3, comparing outcomes between GTR (blue) and STR (orange). Significant differences in survival outcomes are indicated (*p* < 0.001). **C**, **D**: PFS (**C**) and OS (**D**) for WHO Grade 1, showing a clear survival benefit for GTR over STR (*p* < 0.001). **E**, **F**: Progression-free survival (**E**) and overall survival (**F**) for WHO Grade 2. GTR significantly improves PFS (*p* < 0.001), but the difference in OS is not statistically significant (*p* = 0.067). **G**, **H**: PFS (**G**) and OS (**H**) for WHO Grade 3. There is no statistically significant difference between GTR and STR for both PFS (*p* = 0.26) and OS (*p* = 0.27). The number of patients at risk at each time point is shown below each panel, and shaded areas represent 95% confidence intervals

EoR significantly impacted PFS and OS across all grades. Mean PFS was 113.8 months (95%CI: 101.5–126.2) for GTR and 40.1 months (95%CI: 30.7–49.4) for STR (*p* < 0.001, Fig. 3a). Mean OS was 602.9 months (95%CI: 561.4–644.5) for GTR and 173.8 months (95%CI: 152.2–195.5) for STR (*p* < 0.001, Fig. 3b).

### PFS and OS by WHO grades and extent of resection in pediatric meningioma

Stratification by WHO grades revealed that GTR improved PFS and OS across all grades. For WHO grade 1, mean PFS was 243.6 months for GTR vs. 158.7 months for STR (*p* < 0.001, Fig. 3c), with GTR patients showing PFS probabilities of 89.6% at 60 months, 84.1% at 120 and 180 months, compared to 54.6%, 35.6%, and 28.5% for STR. Mean OS was 277.5 months for GTR vs. 177.9 months for STR (*p* < 0.001, Fig. 3d), with OS probabilities of 93.2% at 60 months, 84.9% at 120, and 83.5% at 180 months, vs. 79.8%, 62.4%, and 54.6% for STR.

For WHO grade 2, mean PFS was 128.2 months for GTR vs. 56.1 months for STR (*p* < 0.001, Fig. 3e), with GTR showing PFS probabilities of 100% at 60 months, 67.7% at 120, and 58.0% at 180 months, compared to 40.6% and 25.4% at 60 and 120 months for STR. OS probabilities for GTR were 94.4% at 60 months and 90.7% at 120 and 180 months, compared to 87.5%, 70.1%, and 52.6% for STR (*p* = 0.067, Fig. 3f).

For WHO grade 3, mean PFS was 75.3 months for GTR vs. 56.1 months for STR (*p* = 0.26, Fig. 3g), with GTR showing PFS probabilities of 41.4% at 60 months, 31.1% at 120, and 20.7% at 180 months, compared to 37.1% and 24.7% at 60 and 120 months for STR. Mean OS was 134.8 months for GTR vs. 116.1 months for STR (*p* = 0.27, Fig. 3h), with GTR showing OS probabilities of 93.9% at 12 months, 81.0% at 24, and 72.1% at 60 months, compared to 78.8%, 73.0%, and 66.7% for STR.

### PFS and OS by adjuvant radiotherapy in pediatric WHO grade 2 and 3 meningiomas

**Fig. 4:**
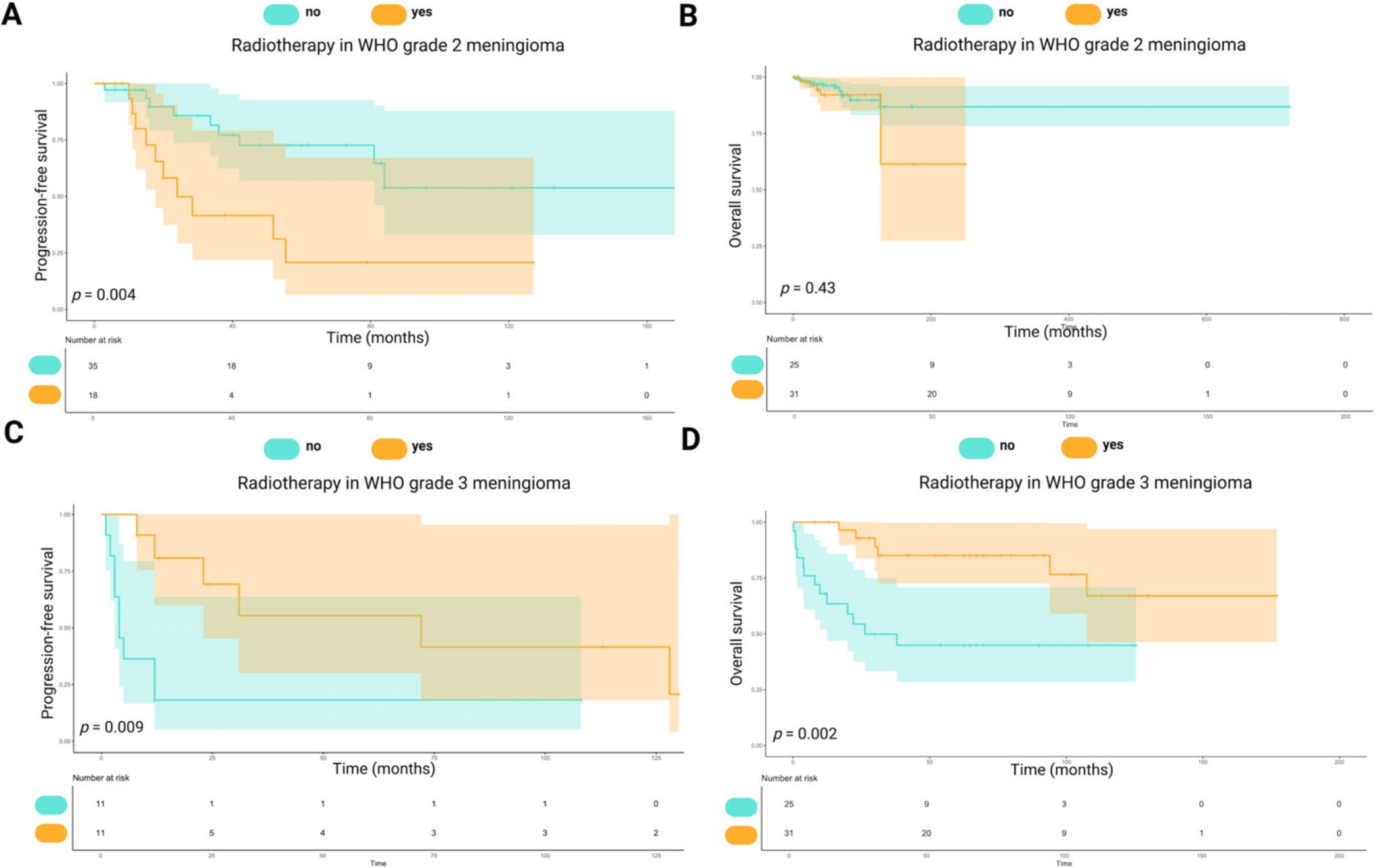
Kaplan–Meier survival analysis for progression-free survival (PFS) and overall survival (OS) based on the use of radiotherapy in WHO Grade 2 and Grade 3 meningiomas. A&B: PFS (**A**) and OS (**B**) for WHO Grade 2 meningiomas, comparing patients who received radiotherapy (orange) versus those who did not (blue). Radiotherapy significantly improved PFS (*p* = 0.004), while no significant difference in OS was observed (*p* = 0.43). **C**, **D**: PFS (**C**) and OS (**D**) for WHO Grade 3 meningiomas, showing a significant improvement in both PFS (*p* = 0.009) and OS (*p* = 0.002) for patients who received radiotherapy. The number of patients at risk at each time point is displayed below each panel, and shaded areas represent 95% confidence intervals

PFS in WHO grade 2 meningiomas was higher without radiotherapy, with 60-month PFS of 72.7% vs. 20.8% with radiotherapy (*p* = 0.004). Mean PFS was 115.2 months (95%CI: 85.5–144.8) without radiotherapy vs. 47.9 months (95%CI: 23.4–72.4) with it (Fig. 4a). OS analysis (56 cases) showed a mean OS of 634.4 months (95%CI: 578.7–690.1) without radiotherapy vs. 194.7 months (95%CI: 132.9–256.5) with it (*p* = 0.43, Fig. 4b).

In WHO grade 3 meningiomas (22 cases), radiotherapy improved outcomes significantly. At 60 months, PFS was 18.2% without radiotherapy vs. 55.4% with it, rising to 41.6% by 100 months (*p* = 0.009, Fig. 4c). Mean PFS was 23.8 months (95%CI: 0.28–47.4) without radiotherapy vs. 72.5 months (95%CI: 38.1–106.9) with it. OS was higher with radiotherapy: mean OS was 63.0 months (95%CI: 39.8–86.2) without radiotherapy vs. 140.7 months (95%CI: 115.8–165.6) with it, with 60-month survival of 44.9% vs. 85.1% (*p* = 0.002, Fig. 4d).

### Subgroup analysis of reconstructed progression-free survival (PFS) data incorporating multiple shared covariates

Subgroup analysis of studies providing IPD with multiple covariates was performed [17–34]. Previous IPD analyses showed a role of adjuvant radiation therapy in aggressive meningiomas. Hence, PFS data of subtotally resected WHO grade 2 and 3 meningiomas was stratified regarding adjuvant radiation therapy. In pediatric WHO grade 1 meningiomas following subtotal resection, the 36-month progression-free survival (PFS) rate for patients receiving adjuvant radiation therapy was 72.2%, compared to 71.3% for those without radiation therapy. At 72 months, the PFS rate for the radiation therapy group declined to 36.1%, while the group without radiation therapy maintained a rate of 63.4%. The results, shown in Part A of Supplementary Fig. 1, indicate no statistically significant difference in PFS between the two groups (*p* = 0.23). The PFS analysis in pediatric WHO grade 2 and 3 meningiomas following subtotal resection with and without adjuvant radiation therapy showed that adjuvant radiation therapy significantly enhanced median PFS time to 55.4 months (95% CI: 25.1–85.7) compared to 5.0 months (95% CI: 0–32.3) for those without radiation (*p* = 0.049). The Kaplan–Meier curve in Supplementary Fig. 1(B) illustrates this difference, highlighting the potential benefit of adjuvant radiation in improving PFS for these higher-grade PMs.

To further investigate prognostic factors regarding PFS in PM, we analyzed 184 of those 1010 patients, who share the following common available covariates: Age, sex, EoR (subtotal resection, gross total resection), and neurofibromatosis status (NF2 or sporadic). We performed uni- and multivariable Cox regression analyses of all factors potentially predicting PFS among these patients separately for each WHO grade to determine independent risk factors of patients sharing common available covariates (see Supplementary Tables 2, 3, 4). In WHO grade 1 meningioma, univariable analysis revealed that STR significantly increased the risk of progression compared to GTR (HR = 7.86, 95% CI: 3.30–18.75, *p* = 0.001), and the absence of adjuvant radiation was similarly associated with poorer PFS (HR = 5.50, 95% CI: 2.30–13.16, *p* = 0.001). Multivariable analysis confirmed only STR as an independent risk factor (HR = 4.51, 95% CI: 1.67–12.25, *p* = 0.003) (see Supplementary Fig. 2). These findings emphasize the importance of achieving GTR in pediatric WHO grade 1 meningiomas. The analyses of PFS in WHO grade 2 and 3 meningiomas were concluded after univariable Cox regression due to the statistical significance of only one variable in each group. For WHO grade 2 meningiomas, STR was the sole significant predictor of worse PFS (HR = 3.57, 95% CI: 1.43–8.93,* p* = 0.007)​. In WHO grade 3 meningiomas, the lack of adjuvant radiation was the only variable associated with significantly poorer PFS (HR = 3.98, 95% CI: 1.28–12.36, *p* = 0.02)​. These results underscore the importance of gross total resection in WHO grade 2 meningiomas and adjuvant radiation in WHO grade 3 meningiomas for improving PFS.

### Bias and quality evaluation

The NIH Quality Assessment Tool revealed most studies had clear objectives, defined populations, adequate recruitment, and measured exposures before outcomes with sufficient follow-up. Limitations included missing sample size justification, unblinded assessors, and limited exposure measure validation, introducing potential bias. Despite this, most studies addressed confounding variables, resulting in a moderate but manageable risk of bias and reliable findings on PMs. The scores for all 14 NIH-QAT domains are summarized in Supplementary Fig. 3.

## Discussion

The present IPD meta-analysis highlights significant differences between PMs and adult meningiomas in the prevalence of more aggressive WHO grades 2 and 3 [34]. While WHO grade 1 accounts for 80–90% of adult meningiomas, grades 2 and 3 are less frequent [1, 7, 35, 36]. In this cohort of 1010 PMs, 44.6% were diagnosed with WHO grades 2 or 3. Key findings include: (1) Time to progression varies across WHO grades, with grade 3 having the poorest PFS. (2) WHO grade 3 shows significantly shorter OS compared to grades 1 and 2, with no OS differences between grades 1 and 2 in children. (3) NF1- and NF2-associated PMs demonstrate significantly shorter PFS than sporadic PMs. (4) EoR impacts OS and PFS in grades 1 and 2, while radiotherapy is recommended particularly for subtotally resected WHO grade 3 PMs. In this meta-analysis of 1010 PM patients, 44.6% were diagnosed with WHO grade 2 or 3 meningiomas. This aligns with previous reports indicating an increased frequency of these higher-grade tumors in pediatric populations [4]. The clear cell subtype is more prevalent in children, contributing to the higher incidence of WHO grade 2 tumors [4]. Classification revisions during the included studies may introduce some bias [37]. Nevertheless, findings from this largest cohort confirm that pediatric WHO grade 3 meningioma patients have significantly shorter PFS and OS compared to grades 1 and 2, with no OS differences between grades 1 and 2.

NF2 alterations, a major driver of PM growth, are over twice as common in children compared to adults [2, 4, 10, 38, 39]. Typical mutations like TRAF7, AKT1, KLF4, SMO, and PIK3CA are rare, while YAP1 fusions in non-NF2-driven PMs promote proliferation and apoptosis [40]. In this study, NF2 prevalence was 10%, with significantly shorter PFS for NF2-positive patients, though OS was unaffected, possibly due to the lower incidence of brain invasion in these cases [6]. NF2-associated meningiomas are often managed less aggressively, balancing treatment risks with disease progression. VEGF receptor vaccines show potential for NF2-associated schwannomas, but their impact on meningiomas needs further investigation [41].

The EoR significantly affects both PFS and OS in PMs, consistent with trends in adults. GTR improved PFS (113.8 vs. 40.1 months) and OS (602.9 vs. 173.8 months) across all grades, emphasizing the importance of complete resection. This aligns with prior pediatric meta-analyses identifying GTR as the strongest predictor of favorable outcomes [5]. GTR benefits persist in WHO grades 2 and 3, though the survival advantage decreases with higher grades.

These findings emphasize that GTR should be prioritized in surgical planning for PMs. Despite GTR, relapse and mortality occurred in some cases, likely due to microscopic brain invasion undetectable during surgery, limitations of postoperative imaging, tumor cell dissemination, multifocal disease, or specific tumor biology contributing to recurrence. These results suggest that while GTR offers benefits regarding tumor control, it does not eliminate the risk of recurrence or death, highlighting the complexity of managing PMs.

Radiotherapy improves PFS and OS in pediatric WHO Grade 3 meningiomas but has no significant impact on all WHO Grade 2 tumors. This paradox may reflect confounding bias, as PMs receiving radiotherapy often have larger residual tumors. Radiotherapy in WHO Grade 3 seems to improve outcomes, and in Grade 2, it is typically reserved for incomplete resections or recurrence, with guidelines recommending a cautious yet proactive approach, weighing long-term risks in pediatric patients [42].

The 2021 WHO classification, integrating molecular markers, has impacted grading and treatment [37]. Many studies lack molecular data, with factors like brain invasion affecting grading. Tumor behavior linked to NF2, CDKN2A/B deletions, and TERT mutations may influence radiotherapy response [43–45]. Pediatric sensitivity to radiation necessitates balancing control and long-term effects. Younger children (< 3 years) may receive 54 Gy in 30 fractions, while older children may receive up to 59.4 Gy [42, 46]. Fractionated radiotherapy minimizes healthy tissue damage, and SRS suits small tumors. High-dose fractionation improves outcomes for WHO Grade 3 while managing neurocognitive risks.

The effectiveness of radiotherapy in completely resected WHO Grade 2 meningiomas remains under investigation (e.g., NRG-BN003, ROAM/EORTC-1308) [47, 48]. Initial results show radiotherapy enhances PFS in immunogenic and NF2-wt meningiomas, with moderate effects for hypermetabolic types and minimal benefits for proliferative cases. Molecular profiling could identify PMs most likely to benefit from adjuvant radiotherapy [49].

### Limitations

This meta-analysis includes IPD collected before the latest WHO classification [37], lacking stratification by markers such as TERT or CDKN2A/B. Multivariate stratification to rule out confounders was not possible in the entire cohort. Selection bias from published data and incomplete datasets may affect results. Limited follow-up may miss late relapses, underestimating recurrence risks, as meningiomas can recur even after 15 years in gross totally resected cases [50].

## Conclusion

Collectively, the present study highlights the significant differences in PFS and OS among PMs based on age, neurofibromatosis, WHO grade, EoR and adjuvant radiotherapy. GTR consistently showed improved PFS and OS across all WHO grades, with a particular survival advantage in WHO Grade 1 and 2 meningiomas. Additionally, adjuvant radiotherapy demonstrated benefits for WHO Grade 3 meningiomas, particularly in subtotally resected PMs. These findings highlight the value of tailored surgical and radiotherapeutic approaches to improve outcomes in PMs and guide future management protocols.

## Supplementary Information

Below is the link to the electronic supplementary material.

Supplementary file1 (PNG 1314 KB)

Supplementary file2 (PNG 879 KB)

Supplementary file3 (PNG 717 KB)

Supplementary file4 (DOCX 27 KB)

Supplementary file5 (DOCX 29 KB)

Supplementary file6 (DOCX 33 KB)

Supplementary file7 (DOCX 16 KB)

Supplementary file8 (DOCX 16 KB)

Supplementary file9 (DOCX 16 KB)

## Data Availability

No datasets were generated or analysed during the current study.
