# Supplementary figures and images for: Overall survival and progression-free survival in pediatric meningiomas: a systematic review and individual patient-level meta-analysis
Source: J Neurooncol. 2025 Jan 9;172(2):289–305. doi: 10.1007/s11060-024-04917-7 (PMC11937060; doi:10.1007/s11060-024-04917-7)

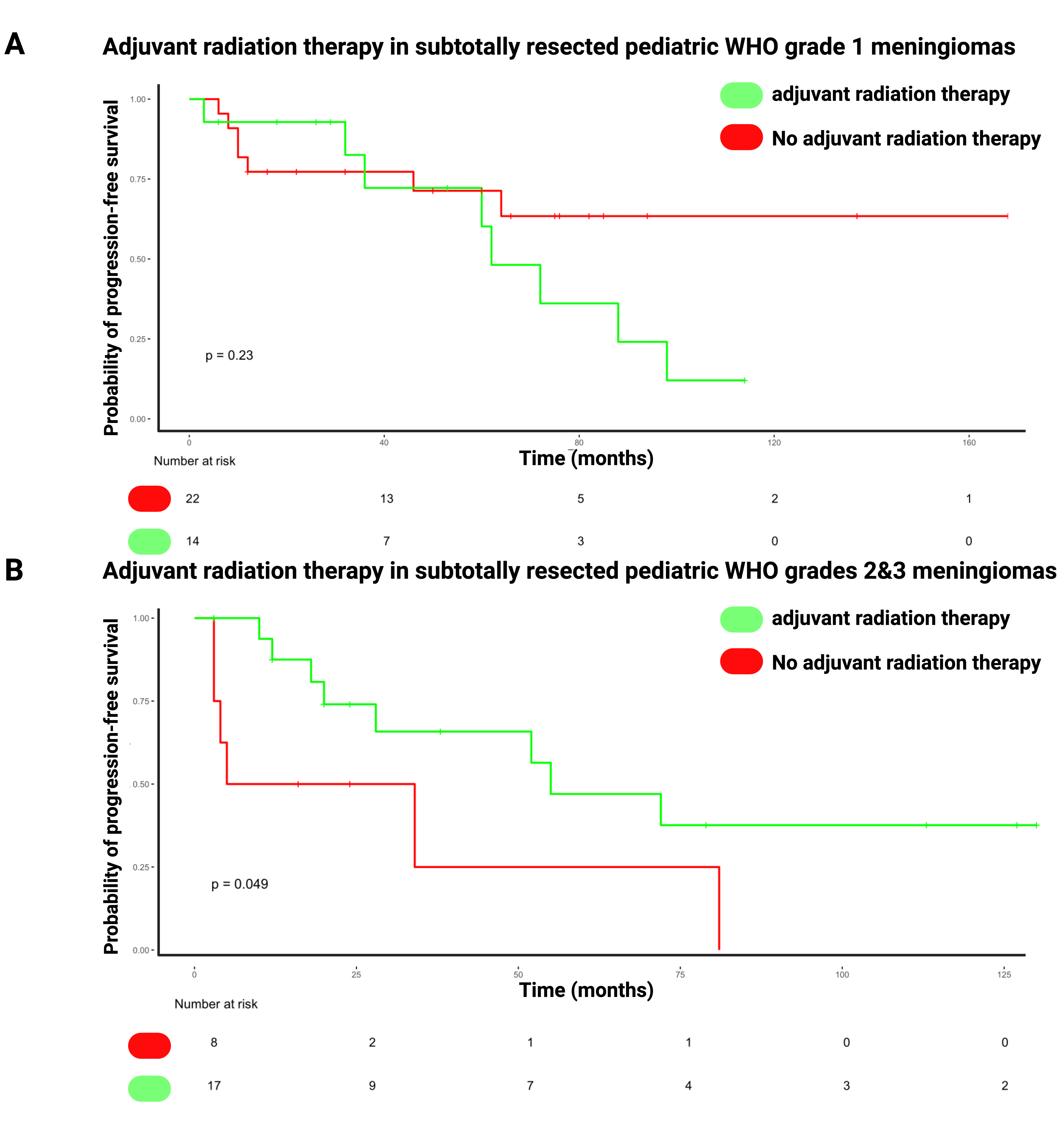

Supplement: Supplementary file 1 — Supplementary file1 (PNG 1314 KB) [file 11060_2024_4917_MOESM1_ESM.png]

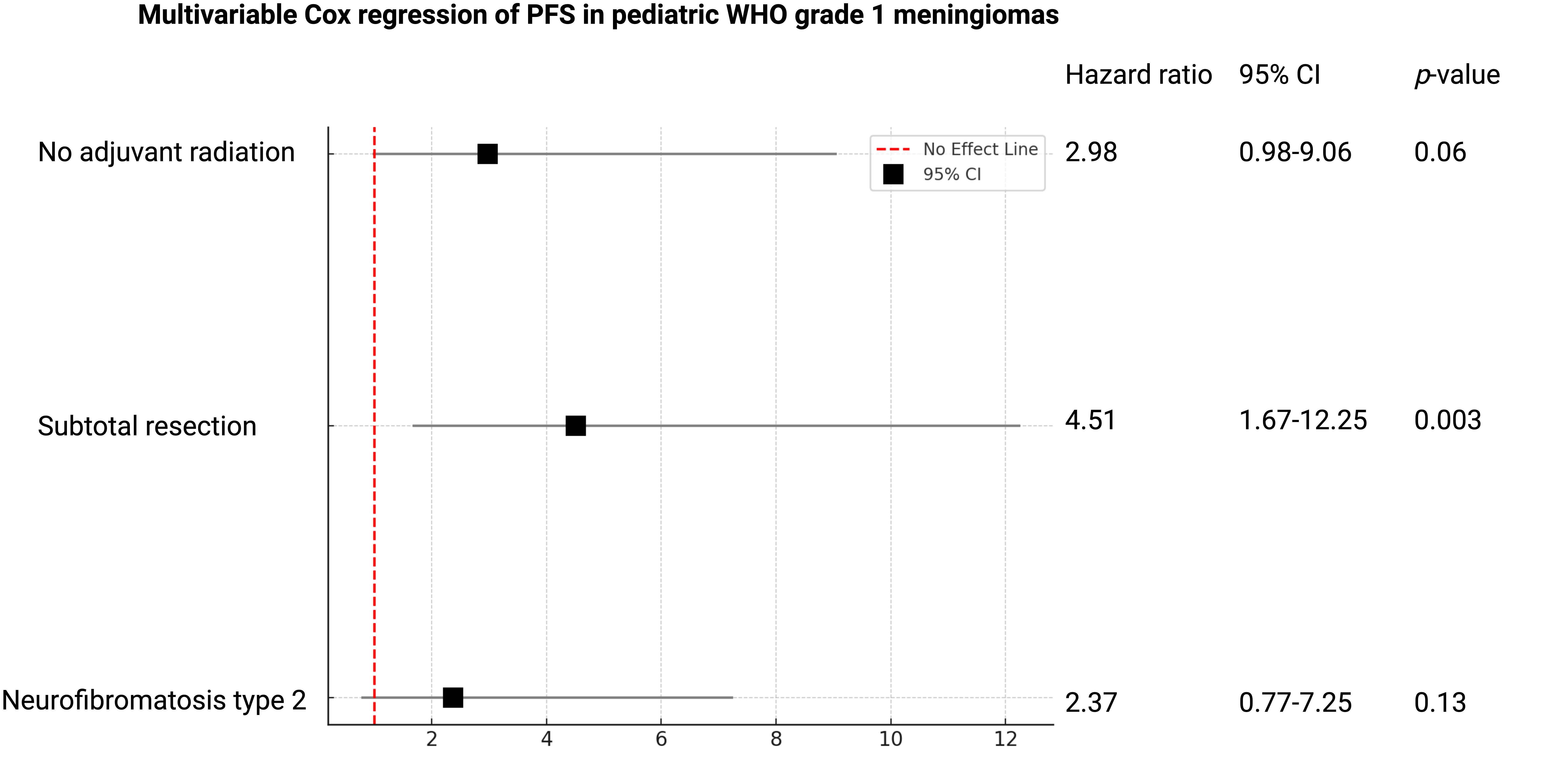

Supplement: Supplementary file 2 — Supplementary file2 (PNG 879 KB) [file 11060_2024_4917_MOESM2_ESM.png]

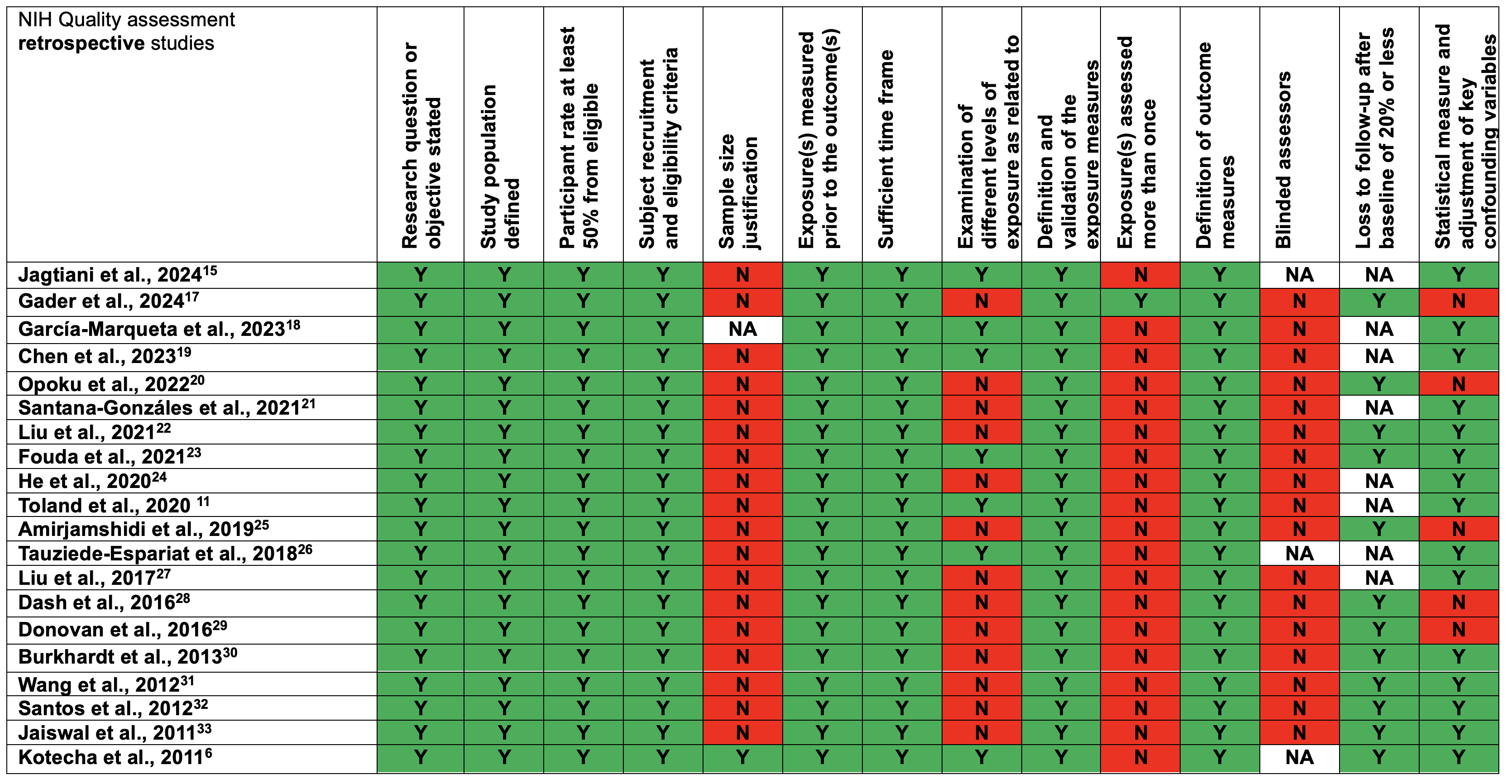

Supplement: Supplementary file 3 — Supplementary file3 (PNG 717 KB) [file 11060_2024_4917_MOESM3_ESM.png]
