# Supplementary material for: Overall survival and progression-free survival in pediatric meningiomas: a systematic review and individual patient-level meta-analysis
Source: J Neurooncol. 2025 Jan 9;172(2):289–305. doi: 10.1007/s11060-024-04917-7 (PMC11937060; doi:10.1007/s11060-024-04917-7)
Supplement: Supplementary file 4 — Supplementary file4 (DOCX 27 KB) [file 11060_2024_4917_MOESM4_ESM.docx]

# Literature Search Summary for Systematic Review on Pediatric Meningiomas

This document provides a comprehensive summary of the literature search conducted across multiple databases to gather relevant studies on pediatric meningiomas. Searches were performed in PubMed, Google Scholar, and the Cochrane Library, focusing on key outcomes such as progression-free survival, overall survival, and other variables such as neurofibromatosis types, resection extent, and the role of adjuvant radiotherapy. The following details outline the search syntax and methodology used in each database.

## 1. PubMed Search

Search Syntax:

(("Meningioma"[Mesh] OR meningioma OR meningiomas) AND ("Child"[Mesh] OR "Adolescent"[Mesh] OR "Infant"[Mesh] OR "Pediatrics"[Mesh] OR childhood OR pediatric OR paediatric OR adolescents OR children OR infants)) AND ("2011/01/01"[Date - Publication] : "3000"[Date - Publication])

## 2. Google Scholar Search

Search Syntax:

"Pediatric Meningioma" OR "Child Meningioma" AND "Progression-Free Survival" OR "Overall Survival" AND "Neurofibromatosis Type 1" OR "Neurofibromatosis Type 2" AND "Extent of Resection" OR "Gross Total Resection"

## 3. Cochrane Library Search

Search Syntax:

("Pediatric Meningioma" OR "Child Meningioma") AND ("Progression-Free Survival" OR "Overall Survival") AND ("Neurofibromatosis Type 1" OR "Neurofibromatosis Type 2") AND ("Extent of Resection" OR "Gross Total Resection") AND "Adjuvant Radiotherapy" AND "WHO Tumor Grade"

.
