# Supplementary material for: Overall survival and progression-free survival in pediatric meningiomas: a systematic review and individual patient-level meta-analysis
Source: J Neurooncol. 2025 Jan 9;172(2):289–305. doi: 10.1007/s11060-024-04917-7 (PMC11937060; doi:10.1007/s11060-024-04917-7)
Supplement: Supplementary file 5 — Supplementary file5 (DOCX 29 KB) [file 11060_2024_4917_MOESM5_ESM.docx]

**Study Protocol**

**Title**:
*Overall Survival and Progression-Free Survival in Pediatric Meningiomas: A Systematic Review and Individual Patient-Level Meta-Analysis*

**Background**

Pediatric meningiomas, while rare, present clinical and biological distinctions from adult cases, making tailored management challenging. ^1,2^ Although they account for a small proportion of central nervous system tumors, pediatric meningiomas differ in histopathological and molecular profiles.^3-5^ This study protocol outlines a systematic review and meta-analysis, aiming to evaluate outcomes like progression-free survival (PFS) and overall survival (OS) by significant factors such as WHO tumor grade, neurofibromatosis (NF) status, extent of resection (EOR), and adjuvant radiotherapy.

**Objectives**

1. To assess OS and PFS in pediatric meningioma patients based on tumor characteristics, including WHO grade and NF status.
2. To analyze the effect of surgical resection extent (gross total vs. subtotal) and the role of adjuvant radiotherapy on survival outcomes.
3. To provide insights into pediatric-specific management guidelines for meningiomas based on survival data.

**Methods**

**1. Eligibility Criteria**

- **Population**: Pediatric patients aged ≤21 years with histopathologically confirmed cranial meningiomas, including NF1 or NF2 associations.
- **Interventions**: Surgical resection categorized into gross total resection (GTR) or subtotal resection (STR), with or without adjuvant radiotherapy.
- **Outcomes**: Primary outcomes are OS and PFS. Secondary analyses include recurrence rates and subgroup differences in survival by treatment.
- **Study Design**: Observational cohort studies, case series with ≥3 patients, and studies reporting on survival data for eligible outcomes are included. Studies should be written in English and only include histopathologically proven meningiomas with WHO grades.

**2. Search Strategy**

- **Databases**: PubMed, Google Scholar, and Cochrane Library.
- **Syntax**:
  - **PubMed**: ("Meningioma"[Mesh] OR meningioma) AND ("Child"[Mesh] OR "Adolescent"[Mesh] OR pediatric) AND ("2011/01/01"[Date - Publication] : "3000"[Date - Publication])
  - **Google Scholar**: "Pediatric Meningioma" OR "Child Meningioma" AND "Progression-Free Survival" OR "Overall Survival" AND "Neurofibromatosis Type 1" OR "Neurofibromatosis Type 2"
  - **Cochrane Library**: ("Pediatric Meningioma" OR "Child Meningioma") AND "Progression-Free Survival" AND "Extent of Resection"
- **Screening and Review**: Independent screening by two reviewers of all titles and abstracts for eligibility, with conflicts resolved by a third reviewer.

**3. Data Extraction**

- **Variables**: Age, sex, neuroanatomical localization, WHO tumor grade, NF1/NF2 status, EOR (GTR or STR), and adjuvant therapy type (e.g., radiotherapy).
- **Extraction Tools**: Data extraction is conducted through standardized forms, with Kaplan-Meier survival data extracted using software like Digitizelt and R’s IPDfromKM package when necessary.^6, 7^ Data consistency checks will be performed by secondary reviewers to ensure accuracy. This reconstruction method will be used for the synthesis of data provided by Kotecha et al^8^. Literature published after this previous meta-analysis was searched and IPD were either directly extracted (if given in full-text or supplementary material) or reconstructed based on Kaplan-Meier curves with number at risk tables. Reconstruction will be also used for the data by Jagtiani et al.^9^.

**4. Study Quality Assessment**

- **Method**: NIH Quality Assessment Tool for Observational Cohort and Cross-Sectional Studies to evaluate methodological rigor and identify potential sources of bias.^10^

**Statistical Analysis**

- **Kaplan-Meier curves** for OS and PFS stratified by WHO grade, NF status, EOR, and adjuvant radiotherapy status.
- **Subgroup comparisons** using log-rank test to determine statistical differences in survival metrics.

**Ethics**

No ethical approval is required as the analysis utilizes only published data. Findings aim to support clinical guideline development.

**Funding and Disclosures**

No external funding was received for this study, and there are no conflicts of interest.

**References**

1. QT, Price M, Neff C, Cioffi G, Waite KA, Kruchko C, Barnholtz-Sloan JS. CBTRUS Statistical Report: Primary Brain and Other Central Nervous System Tumors Diagnosed in the United States in 2016-2020. Neuro Oncol. 2023 Oct 4;25(12 Suppl 2):iv1-iv99. doi: 10.1093/neuonc/noad149.
2. Sahm F, Schrimpf D, Stichel D, Jones DTW, Hielscher T, Schefzyk S, Okonechnikov K, Koelsche C, Reuss DE, Capper D, Sturm D, Wirsching HG, Berghoff AS, Baumgarten P, Kratz A, Huang K, Wefers AK, Hovestadt V, Sill M, Ellis HP, Kurian KM, Okuducu AF, Jungk C, Drueschler K, Schick M, Bewerunge-Hudler M, Mawrin C, Seiz-Rosenhagen M, Ketter R, Simon M, Westphal M, Lamszus K, Becker A, Koch A, Schittenhelm J, Rushing EJ, Collins VP, Brehmer S, Chavez L, Platten M, Hänggi D, Unterberg A, Paulus W, Wick W, Pfister SM, Mittelbronn M, Preusser M, Herold-Mende C, Weller M, von Deimling A. DNA methylation-based classification and grading system for meningioma: a multicentre, retrospective analysis. Lancet Oncol. 2017 May;18(5):682-694. doi: 10.1016/S1470-2045(17)30155-9.
3. Nassiri F, Liu J, Patil V, Mamatjan Y, Wang JZ, Hugh-White R, Macklin AM, Khan S, Singh O, Karimi S, Corona RI, Liu LY, Chen CY, Chakravarthy A, Wei Q, Mehani B, Suppiah S, Gao A, Workewych AM, Tabatabai G, Boutros PC, Bader GD, de Carvalho DD, Kislinger T, Aldape K, Zadeh G. A clinically applicable integrative molecular classification of meningiomas. Nature. 2021 Sep;597(7874):119-125. doi: 10.1038/s41586-021-03850-3.
4. Tauziède-Espariat A, Pfister SM, Mawrin C, Sahm F. Pediatric meningiomas: A literature review and diagnostic update. Neurooncol Adv. 2023 Jun 3;5(Suppl 1):i105-i111. doi: 10.1093/noajnl/vdac165.
5. Ostrom QT, Gittleman H, Fulop J, Liu M, Blanda R, Kromer C, Wolinsky Y, Kruchko C, Barnholtz-Sloan JS. CBTRUS Statistical Report: Primary Brain and Central Nervous System Tumors Diagnosed in the United States in 2008-2012. Neuro Oncol. 2015 Oct;17 Suppl 4(Suppl 4):iv1-iv62. doi: 10.1093/neuonc/nov189.
6. Rakap S, Rakap S, Evran D, Cig O. Comparative evaluation of the reliability and validity of three data extraction programs: UnGraph, GraphClick, and DigitizeIt. Comput Hum Behav. 2016;55:159-66. doi: 10.1016/j.chb.2015.09.008.
7. Liu N, Zhou Y, Lee JJ. IPDfromKM: reconstruct individual patient data from published Kaplan-Meier survival curves. BMC Med Res Methodol. 2021 Jun 1;21(1):111.
8. Kotecha RS, Pascoe EM, Rushing EJ, Rorke-Adams LB, Zwerdling T, Gao X, Li X, Greene S, Amirjamshidi A, Kim SK, Lima MA, Hung PC, Lakhdar F, Mehta N, Liu Y, Devi BI, Sudhir BJ, Lund-Johansen M, Gjerris F, Cole CH, Gottardo NG. Meningiomas in children and adolescents: a meta-analysis of individual patient data. Lancet Oncol. 2011 Dec;12(13):1229-39. doi: 10.1016/S1470-2045(11)70275-3.
9. Jagtiani P, Karabacak M, Le C, Bahadir Z, Morgenstern P, Margetis K. Comprehensive assessment of atypical and anaplastic pediatric meningiomas utilizing national cancer database: a retrospective cohort study. Childs Nerv Syst. 2024 Aug;40(8):2345-57. doi: 10.1007/s00381-024-06431-7. Epub 2024 May 9. PMID: 38722323.
10. Ma LL, Wang YY, Yang ZH, Huang D, Weng H, Zeng XT. Methodological quality (risk of bias) assessment tools for primary and secondary medical studies: what are they and which is better? Mil Med Res. 2020;7(1):7. doi: 10.1186/s40779-020-00238-8.


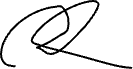


Priv.-Doz. Dr. med. Johannes Wach, MBA

Department of Neurosurgery

University Hospital Leipzig

Leipzig University

Liebigstraße 20, 04103 Leipzig, Germany
