# Supplementary material for: Overall survival and progression-free survival in pediatric meningiomas: a systematic review and individual patient-level meta-analysis
Source: J Neurooncol. 2025 Jan 9;172(2):289–305. doi: 10.1007/s11060-024-04917-7 (PMC11937060; doi:10.1007/s11060-024-04917-7)
Supplement: Supplementary file 6 — Supplementary file6 (DOCX 33 KB) [file 11060_2024_4917_MOESM6_ESM.docx]

**Supplementary table 1:** PRISMA Checklist for Systematic Review and Meta-Analysis on Pediatric Meningiomas

| Section | Item | Description | Page Number |
| --- | --- | --- | --- |
| Title | Title | Overall survival and progression-free survival in pediatric meningiomas: A systematic review and individual patient-level meta-analysis | 1 |
| Abstract | Structured Summary | Structured abstract summarizing Background, Objectives, Data Sources, Eligibility Criteria, Study Appraisal, Synthesis Methods, Results, Limitations, Conclusions, and clinical implications is given. | 3 |
| Introduction | Rationale | Need for large-scale pediatric meningioma research and addressing the gap in adult-derived management guidelines. | 4 |
| Introduction | Objectives | Targeted outcomes like overall survival (OS) and progression-free survival (PFS) by age, WHO grade, extent of resection, adjuvant radiotherapy, and neurofibromatosis status. | 4-5 |
| Methods | Protocol and registration | The systematic review was prospectively registered in the International Prospective Register of Systematic Reviews (PROSPERO, Registration ID: CRD42024601057) | 5 |
| Methods | Eligibility Criteria | Inclusion criteria, focusing on patient age (≤21 years), histopathologically confirmed meningioma diagnosis with WHO grade, and availability of PFS or OS data. | 5 |
| Methods | Information Sources | List all sources searched, such as PubMed, with search dates from January 1, 2011, to August 30, 2024. Furthermore Google Scholar and Cochrane library were searched. Individual patient data (IPD) was reconstructed from the previous largest meta-analysis. | 5 |
| Methods | Search Strategy | Detail search strategy including keywords and MeSH terms like “meningioma,” “pediatric,” and “neurofibromatosis.” | 5 |
| Methods | Selection Process | Two reviewers independently search and screened literature for selection. Discrepancy were checked by a third reviewer. | 5 |
| Methods | Data Collection Process | Digitezit and the R package IPDfromKM were used for individual patient data (IPD) on key variables such as age, sex, tumor grade, and treatment factors (e.g., extent of resection, use of radiotherapy). | 5 |
| Methods | Data Items | Patient & tumor characteristics (e.g., Age, Neurofibromatosis status, extent of resection, WHO grade, adjuvant radiotherapy) | 5 |
| Methods | Study Risk of Bias Assessment | Risk of bias evaluation using the NIH Quality Assessment Tool for Observational Cohort and Cross-Sectional Studies (NIH-QAT) was performed. | 5 |
| Methods | Effect Measures | Kaplan-Meier survival estimates stratified by age, tumor WHO grade, extent of resection, adjuvant radiotherapy and neurofibromatosis status. PFS and OS rates were calculated | 5 |
| Methods | Synthesis of Results | Subgroup analyses for OS and PFS based on age, WHO grade, neurofibromatosis status, adjuvant radiotherapy and extent of resection. | 5 |
| Methods | Risk of Bias Across Studies | Summary of risk of bias across studies based on NIH-QAT findings is given. | 5 |
| Results | Study Selection | Provide number of studies screened, assessed for eligibility, and included in the review, along with reasons for exclusions (e.g., lack of PFS data). | 14 |
| Results | Study Characteristics | Describe key characteristics of each study, such as location, sample size, patient demographics, and study design. | 14 |
| Results | Results of Individual Studies | Summarize outcomes for OS and PFS across individual studies, stratified by factors like extent of resection and WHO tumor grade. | 14 |
| Results | Synthesis of Results | Report pooled results, including Kaplan-Meier estimates for OS and PFS by age, tumor grade, neurofibromatosis status, and resection extent. | 15-22 |
| Results | Risk of Bias in Studies | Summarize findings from the NIH-QAT assessment on study quality and bias, including factors like sample size justification and blinding. | 22 |
| Discussion | Summary of Evidence | Provide an overview of key findings, emphasizing the survival impacts of gross total resection (GTR) and radiotherapy on pediatric meningiomas. | 23-25 |
| Discussion | Limitations | Discuss limitations such as potential sample size bias, lack of molecular data, and limited applicability of some adult meningioma protocols. | 25 |
| Discussion | Conclusions | State the clinical implications, emphasizing the need for pediatric-specific guidelines in meningioma treatment. | 25 |
| Funding | Funding | Mention the study did not receive external funding. | 2 |
| Acknowledgments | Contributions | Acknowledge any additional contributions by authors and creation tools (e.g., BioRender for visual abstracts). | 26 |
