# Supplementary material for: Overall survival and progression-free survival in pediatric meningiomas: a systematic review and individual patient-level meta-analysis
Source: J Neurooncol. 2025 Jan 9;172(2):289–305. doi: 10.1007/s11060-024-04917-7 (PMC11937060; doi:10.1007/s11060-024-04917-7)
Supplement: Supplementary file 7 — Supplementary file7 (DOCX 16 KB) [file 11060_2024_4917_MOESM7_ESM.docx]

| Supplementary table 2. Univariable Cox regression analysis of progression-free survival in pediatric WHO grade 1 meningiomas | | | |
| --- | --- | --- | --- |
| Variable | Univariable | | |
|  | HR | 95% CI | *p*-Value |
| Age | 1.03 | 0.50–2.10 | 0.95 |
| (**≤11** vs. >11) |  |  |  |
| Sex (**male**/female) | 1.26 | 0.62-2.55 | 0.53 |
| Extent of resection  (**subtotal resection** vs. gross total resection) | 7.86 | 3.30–18.75 | 0.001 |
| Adjuvant radiation (**No adjuvant radiation** vs. adjuvant radiation) | 5.50 | 2.30–13.16 | 0.001 |
| Neurofibromatosis type 2 (**Present** vs. Absent) | 2.12 | 0.92-4.92 | 0.08 |
| CI, Confidence Interval; HR, Hazard Ratio | | | |
