# Supplementary material for: Overall survival and progression-free survival in pediatric meningiomas: a systematic review and individual patient-level meta-analysis
Source: J Neurooncol. 2025 Jan 9;172(2):289–305. doi: 10.1007/s11060-024-04917-7 (PMC11937060; doi:10.1007/s11060-024-04917-7)
Supplement: Supplementary file 8 — Supplementary file8 (DOCX 16 KB) [file 11060_2024_4917_MOESM8_ESM.docx]

| Supplementary table 3. Univariable Cox regression analysis of progression-free survival in pediatric WHO grade 2 meningiomas | | | |
| --- | --- | --- | --- |
| Variable | Univariable | | |
|  | HR | 95% CI | *p*-Value |
| Age | 1.05 | 0.47–2.35 | 0.90 |
| (**≤11** vs. >11) |  |  |  |
| Sex (**male**/female) | 1.25 | 0.54-2.93 | 0.60 |
| Extent of resection  (**subtotal resection** vs. gross total resection) | 3.57 | 1.43–8.93 | *0.007* |
| Adjuvant radiation (**No adjuvant radiation** vs. adjuvant radiation) | 1.82 | 0.77–4.29 | 0.17 |
| Neurofibromatosis type 2 (**Present** vs. Absent) | 1.42 | 0.47-4.29 | 0.54 |
| CI, Confidence Interval; HR, Hazard Ratio | | | |
